# Observations on Some Medicinal Products of Australian Plants

**Published:** 1832-10

**Authors:** 


TRANSACTIONS OF THE MEDICO-BOTANICAL SOCIETY OF LONDON,

*Published under the Direction of the President and Council.*

### AUSTRALIAN PLANTS.

### *Observations on some Medicinal Products of Australian Plants.* By Mr. Mudie.

The novel and peculiar botany of the great southern continent of Australia has not been examined so minutely, with reference to the additions that it may make to the materials of the healing art, as might, from its importance, be expected. Several causes conspire to produce this; of these, one is general, and applies to all newly-discovered countries alike, and others arise from the peculiarities of that particular country. The vast improvements which were made in chemistry, more especially during the last century, had a natural tendency to mineralize the Pharmacopœia, and to take off the attention of inquiring minds from the medical properties of vegetables. It is not easy to say how much has been gained on the one side or lost on the other; but that there has been loss, is certain; and those who wish that the resources of all the kingdoms of nature should be equally open to man cannot fail to regard, in the most grateful and favorable manner, the labours of the Society instituted for the express purpose of doing justice to the medical qualities of plants.

The peculiar causes are to be found partly in the limited knowledge that we have of Australia, partly in the dispersion of its vegetables, partly in their uniformity, and partly in the ease with which the colonies are supplied with medicines from the mother country. There is another cause, the ignorance of the natives when first visited. It does not appear that their medicines extended beyond chewed or pounded grass, a poultice of mud, or fomentation produced by the application of a broiled fish. Of course, they could not point out medicinal plants to the European visiters; while these have hitherto been too constantly occupied in erecting habitations and finding food, for investigating the virtues of plants with that patience and nicety which a subject so very recondite demands.

The principal Australian substances which present themselves to the inquiries of the medical botanist, are chiefly gums, or resins, of some description or other, and oils. The oils are chiefly obtained from the genus Melaleuca, the species of which are very numerous, and some of them are understood to yield an oil resembling in its virtues the cajeput oil of the south-east of Asia. The gums and resins are chiefly obtained from the stem of Xanthorrhœa, and from exudations upon the bark of Acacia and Eucalyptus. The latter is a very abundant genus, and the gum which it affords is astringent, like kino or myrrh; while that of the Acacia is more mucilaginous, and approaches nearer to gum arabic or the gums of the genus Prunus, as common in this country. One species of Eucalyptus affords a substance, of which the medicinal properties have been tried in Australia, and which, should it be found to suffer no injury or decomposition during the voyage, might materially reduce the price of manna, which still retains its rank as a medicine.

Until the substance itself, which appears rather difficult to preserve, be laid before the Society for their examination and decision, it may not be altogether useless to offer a few notes on the plant, as even that is but imperfectly known, and has not, it is believed, been figured or described in any scientific work. The following are the characters and a few points in the description:

*Generic character of Eucalyptus.* Calyx superus, persistens, truncatus ante anthesin tectus. Operculo integerrimo, deciduo. Corolla nulla. Capsula quadrilocularis, apice dehiscens, polysperma.

*Natural order:* Myrtaceæ.

*Specific name:* Eucalyptus mannifera. (Manna gum tree.)

*Specific character:* Operculo hemisphærico acutiusculo, umbellis axillaribus terminalibusve 4-6 floris, cortice albo-cinereo.

The principal habitat of this tree is upon the elevated downs into which the Blue Mountains subside, and upon the adjoining slopes. It grows to the height of from thirty to forty feet, of irregular growth, and having a number of slender branches. As is the case with almost all the genus, the leaves, which are simple, lanceolate, and entire, are placed vertically, by a peculiar twist of the footstalk.

From the accounts given of it, it does not appear that the manna produced by this Eucalyptus is very different from that yielded by the Fraxinus, on the coast of the Mediterranean; though, as common report describes it as having less of the nauseous taste, it may be less efficient as a medicine. Like the manna of Europe, it is reported to contain a saccharine and a mucous ingredient, both of which are easily soluble in water, and partially so likewise in the atmosphere, when moist. It obviously arises from a rupture in the cortical vessels of the tree, produced not by the puncture of insects, but by atmospheric action, as it is produced only in the dry season, and the quantity varies with the degree and duration of the drought.

Toward the close of a long dry season, it is found so abundant on the ground under the trees, that several pounds may be collected by one person in a few minutes; but when rain begins to fall, it melts, and disappears almost as rapidly as snow.

It is worthy of remark, that this substance, manna, which is reported as being so similar, as afforded by the Ash of Europe and the Eucalyptus of Australia, and which has not been found, possessing the same qualities, upon any other species of plant, should yet appear to be an exudation of two genera which differ so much in every other respect, and are indigenous only in countries which are distant from each other by nearly half the circumference of the globe; and it deserves further notice, as being one of the few instances of near coincidence in a substance where most of the productions of nature are so dissimilar.

